# Disability research in African Network for Evidence-to-Action in Disability affiliated countries

**DOI:** 10.4102/ajod.v13i0.1517

**Published:** 2024-11-08

**Authors:** Callista K. Kahonde, Gubela Mji

**Affiliations:** 1 Department of Global Health, Faculty of Medicine and Health Sciences, Stellenbosch University, Cape Town, South Africa

**Keywords:** disability research, Africa, African Network for Evidence-to-Action in Disability, AfriNEAD, collaborations

## Abstract

**Background:**

The African Network for Evidence-to-Action in Disability (AfriNEAD) is a leading role player in Africa promoting evidence-informed policies and practices for disability inclusion on the continent. This article presents findings of a desktop review that explored trends of disability research in the AfriNEAD affiliated countries.

**Objectives:**

The review explored trends of research that has been published by members of the disability research community who are contributing to AfriNEAD Conferences.

**Method:**

A Google scholar search was conducted using names of researchers who presented articles at the first six AfriNEAD Conferences, recording peer-reviewed journal publications by each author according to the eight AfriNEAD research focus areas. This was followed by a hand search of all articles published in the *African Journal of Disability* from AfriNEAD affiliated countries.

**Results:**

There is an exponential increase in the number of peer reviewed journal publications from AfriNEAD affiliated countries over the last two decades. Collaborations are common among authors within the same African country. International collaborations are common among authors from Africa with authors from the Global North.

**Conclusion:**

African researchers need to network and collaborate more across Africa, to promote disability research in countries where research is scarce and to focus more on research areas that are not receiving attention.

**Contribution:**

The desktop exploration is a first step for AfriNEAD to get a baseline understanding of published disability research in the countries affiliated to the network. Further research is required to understand these trends and to provide evidence necessary to address the identified gaps.

## Background

Persons with disabilities experience marginalisation and discrimination globally (Grue 2019; World Health Organization [WHO] 2022). The advent of disability rights and advocacy discourses and frameworks such as the United Nations Convention on the Rights of Persons with Disabilities (UNCRPD), which is the major treaty that guides disability practice in the world, has raised society’s consciousness of the need for disability-inclusive practices and policy frameworks (United Nations [UN] 2006). However, this shift still needs to be supported by more research evidence to inform policies and their accompanying practices globally and more so on the African continent where disability has been at the bottom rung of the development agenda (Grech & Soldatic 2016).

The African Network for Evidence-to-Action in Disability (AfriNEAD) is a prominent role player on the continent, working to promote evidence-informed policies and practices for disability inclusion in Africa (Kachaje et al. 2014). African Network for Evidence-to-Action in Disability, based at the Division of Disability and Rehabilitation Studies (DDRS) at Stellenbosch University, was incepted in November 2007. The network seeks solutions to remove obstacles and barriers faced by persons with disabilities. It focusses on bridging the practical ‘know-how’ of persons with disabilities and research evidence by bringing together a broad range of relevant stakeholders to facilitate translation of disability research evidence into policy and practice for the realisation of the rights of persons with disabilities in the African continent. It uses networking as a channel to facilitate sharing and development of disability research among researchers and persons with disabilities on the continent. We refer the reader to past articles that outline the foundations of the network for more information on the history of the network (Kachaje et al. 2014; Mji et al. 2009, 2011).

At its core, AfriNEAD strives to promote the realisation of the UNCRPD principles in Africa through its four main pillars, namely, triennial conferences, the *African Journal of Disability* (AJOD), ongoing networking and disability research country working groups (DRCWGs; see AfriNEAD website: https://blogs.sun.ac.za/afrinead/). This article presents findings of an exploratory desktop study that was conducted to establish the trends of disability research in countries that are affiliated^1^ to AfriNEAD. The countries include Cameroon, Lesotho, Democratic Republic of Congo (DRC), South Africa, Malawi, Ghana, Zimbabwe, Botswana, Ethiopia, Kenya, Namibia, Nigeria, Rwanda, Sierra Leone, Sudan, Tanzania, Uganda and Zambia. Before presenting the focus of the study and its results, a snapshot of the different activities of the network, which are closely related to the research activities, is presented.

### African Network for Evidence-to-Action in Disability triennial conferences

African Network for Evidence-to-Action in Disability tables conferences every 3 years where academic researchers and other relevant stakeholders come together to share evidence from their research activities and deliberate on practical ways by which the research evidence can be used to promote the rights of persons with disabilities in Africa. At the time of conducting this desktop study, the network had managed to table six^2^ conferences since its inauguration in 2007. The presentations and discussions were guided by eight research areas that the network established and refined over the 17 years of its existence. The Articles of the UNCRPD (UN 2006) were used as the guiding framework to establish these eight research areas pertinent to the African context. These areas are not directly linked to each specific Article of the UNRCPD, but they were identified as themes that the network can use to address the different UNCRPD Articles. The eight areas are listed as follows:

- Assistive Technology.
- Children and Youth with Disabilities.
- Systems of Community-Based Rehabilitation (CBR).
- Development Process in Africa: Poverty, Politics, and Indigenous Knowledge systems.
- Economic Empowerment.
- Education: Early Childhood to Tertiary.
- Health and human immunodeficiency virus (HIV) and acquired immunodeficiency syndrome (AIDS).
- Holistic Wellness: Sport, Recreation, Sexuality and Spirituality.

### The African Journal of Disability

A need for a platform to publish research presented at each conference and other research conducted in Africa was identified at the 2009 conference. African Network for Evidence-to-Action in Disability then partnered with Kwame Nkrumah National University of Technology (KNUST) and the DDRS Stellenbosch University to establish the AJOD. The journal has since become an important vehicle for sharing research evidence among African scholars and others from various regions interested in advancing disability research in Africa. African Network for Evidence-to-Action in Disability successfully publishes an issue every year since 2012, and it is now indexed in more than 10 databases. Following each conference, a special issue dedicated to the conference is published and the special issue editorials present an overview of the conference proceedings and the outcome and resolutions in terms of research evidence.

### Networking among disability researchers in Africa and partners in other regions

Members of AfriNEAD have established working relationships and they engage in ongoing networking in between the conferences and at the conferences. Networks formed at conferences sometimes contribute to research and advocacy collaborations. The secretariat sends regular updates on the network’s activities via a monthly newsletter and meets with members of Disability Research Country Working Groups (DRCWGs) on a regular basis. Between conferences, there is ongoing networking between disability researchers, organisations for persons with disabilities (OPDs) and civil society on how disability research evidence can be better translated into policy to enhance the well-being of persons with disabilities.

### Disability research country working groups

Disability research country working groups are local groups within AfriNEAD-affiliated countries that mimic the concept of the main network by bringing together disability researchers and other stakeholders to promote disability research and its implementation within their country. The DRCWGs are tasked with packaging the evidence and recommendations from the conferences as relevant to their local contexts and also work on identifying evidence gaps in their countries as well as potential role players in disability research and policy implementation. They receive regular support from the AfriNEAD secretariat and get space to share the status of disability research in their countries at the triennial conferences and to network with other groups. At the time of writing this manuscript, there are eight DRCWGs that have been established. These are in the Democratic Republic of Congo, Ghana, Kenya, South Africa, Tanzania, Uganda, Zambia and Zimbabwe.

Through all the activities described earlier, AfriNEAD is a leading role player in Africa, promoting the establishment of strong networks and conducting of research and its implementation to ultimately achieve inclusion and realisation of human rights by persons with disabilities. Understanding of research trends enables the network and its members to see developments and progress in different countries and across the region and identify gaps that the disability research community needs to focus on.

## Methods

### Data collection

Data collection was conducted from April to June 2023, following two criteria. Firstly, a hand search of names of *all* the authors who presented at AfriNEAD Conferences since its inception in 2007 was conducted. These names were retrieved from the six conferences’ abstract booklets. Articles published by these authors were then retrieved from Google Scholar while recording each article on a Microsoft (MS) Excel spreadsheet. The details recorded on the spreadsheet were author name and affiliation, year of publication and country of publication or where the study was conducted. Secondly, a hand search of all the articles published in the AJOD from AfriNEAD-affiliated countries was conducted, starting with the first issue published in 2012 up to June 2023 publications. Data were recorded on the same spreadsheet as in the initial phase of data collection. For both stages of data collection, some articles included were written by authors outside Africa, but the research was conducted in Africa, in one of the AfriNEAD-affiliated countries.

For an article to be included to the list, it had to be based on research conducted in at least one of the AfriNEAD-affiliated countries or written by authors from at least one of these countries and had to be published in English. The articles also had to have disability as an issue of human rights, inclusion and social justice as its main focus and not focussed solely on medical and/or therapeutic interventions and practices for persons with disabilities. The study was an exploratory exercise for AfriNEAD to get a baseline understanding of the research trends and did not follow specific standards for reviews. The nature of the study also did not require it to go through ethics approval.

The data were categorised into eight MS Excel workbooks according to the eight research focus areas developed by AfriNEAD, which were listed earlier. Although there are overlaps across the different research focus areas, an article was classified according to what was deemed as the main area of focus of the research. For example, an article could be about children and youth but with a main focus of access to education. Such an article would be placed under education.

### Data analysis

Duplicates were removed before analysis. The articles in each research focus area were analysed using three themes, namely, *the country or region where the studies were conducted, years of publication* and *author affiliations*. The sort and filter function in MS Excel was used to sort the articles and count number of articles in each category; for example, when looking at year of publication, the number of articles published in each year was filtered for each of the eight focus areas and totals recorded.

## Results

The first phase of data collection revealed that there are many researchers who present at AfriNEAD Conferences, but their research is not published in peer-reviewed journals as the name searches did not retrieve publications by many of these researchers. In this section, the results are presented under each of the eight focus areas using tables as shown in the following section.

### Theme 1: Country/region of publication

Table 1 shows that South Africa has produced a disproportionate number of articles when compared to all the other countries affiliated to AfriNEAD. This is the case across all the focus areas. Countries like Ghana, Malawi, Zambia and Zimbabwe follow behind South Africa, although their total number of publications are still less than a quarter of the contribution from South Africa. The region named LMIC covers articles that had a focus on low- and low-middle income countries with others outside Africa and the region named Africa refers to a focus spanning across multiple African countries.

**TABLE 1:** Distribution of articles according to country/region of publication.

Research area	Country/region																					
	Botswana	Cameroon	DRC	Ethiopia	Ghana	Kenya	Lesotho	Malawi	Namibia	Nigeria	Rwanda	Sierra Leone	Sudan	South Africa	Tanzania	Uganda	Zambia	Zimbabwe	LMIC	Africa	Review (global)	Totals
Assistive technology	1	0	0	0	1	1	0	7	0	0	0	0	0	14	1	2	0	2	0	5	12	**46**
Children and youth	0	1	0	2	6	3	0	6	1	1	0	0	0	39	1	2	7	6	3	3	3	**84**
Systems of CBR	0	1	0	1	2	0	0	0	3	1	1	0	0	50	2	4	2	0	0	6	0	**73**
Development processes	2	3	1	1	12	1	4	13	1	4	0	1	2	58	1	6	10	8	1	29	0	**158**
Economic empowerment	1	0	1	0	4	2	0	1	0	0	0	1	0	37	0	1	1	2	1	1	0	**53**
Education	2	4	0	0	16	6	2	5	0	3	0	0	0	102	1	3	21	20	0	4	0	**189**
Health and HIV and AIDS	3	5	0	1	10	0	0	9	3	0	1	0	0	65	0	3	4	7	0	13	4	**128**
Holistic wellness	0	0	0	1	4	0	0	0	0	1	1	0	0	47	0	2	2	8	1	0	0	**67**

**Totals**	**9**	**14**	**2**	**6**	**55**	**13**	**6**	**41**	**8**	**10**	**3**	**2**	**2**	**412**	**6**	**23**	**47**	**53**	**6**	**61**	**19**	**798**

*Assistive Technology* has a wide range of geographical spread in terms of the country or region where research was conducted and/or published. Unlike the other research focus areas, more articles focussed on more than one country and approached the area of *Assistive Technology* with a broader and global perspective. Only just about a third of the articles came from a single African country (Botswana, Ghana, Kenya, Lesotho, Malawi, Tanzania, Uganda, Zimbabwe) followed by those published with a focus spanning multiple continents.

The focus area of *Children and Youth* has articles published from a wide range of African countries. More than half of these articles came from a single African country (Cameroon, Ethiopia, Ghana, Kenya, Malawi, Namibia, Nigeria, South Africa, Tanzania, Uganda, Zambia, Zimbabwe), followed by a few that had an LMIC or African focus or were systematic reviews. Articles published on *Systems of CBR* are mostly based on research conducted in a single African country, with a few multi-country studies that are mostly reviews.

*Development Processes in Africa* is one of the focus areas with the highest number of publications retrieved with a total of 158, second to *Education* with a total of 189. Countries like Zambia, Malawi and Ghana have at least 10 publications per each country under *Development Processes in Africa*, which is unusual in other focus areas, although they are way behind South Africa, which has a high total of 58. Research on *Economic Empowerment* is not popular in the retrieved publications. Multiple country studies are also scarce under this focus area.

*Education* with its 189 articles is the most popular focus area, with more than triple the number of publications in other focus areas. South Africa also contributes a disproportionately high number of publications in this area, with Zambia and Zimbabwe trailing behind South Africa with at least 20 articles each. The focus area of *Health and HIV and AIDS* has more articles focussing on multiple countries in Africa than in other areas, coming second after *Development Processes in Africa* in this row.

The research focus area of *Holistic Wellness* mostly shows publications from a single African country (Ethiopia, Ghana, Nigeria, Rwanda, South Africa, Uganda, Zambia, Zimbabwe), with no articles from countries like Malawi and Cameroon, which are popular in other focus areas. Generally, the focus area also tends to have fewer publications in comparison with the other areas.

### Theme 2: Year of publication

Publications started surfacing since the late eighties, and the numbers for a few of the focus areas have continued to rise, especially after 2006. The last phase that the review focussed on from January 2021 to June 2023 also showed a high number of articles across most of the focus areas, although the period is only two and half years. A total of 218 articles were recorded during this two-and-a-half-year period.

Published *Assistive Technology* research happens to be a late comer on the disability research platform in AfriNEAD-affiliated countries, with the first article identified having been published in 2013. Since then, there has been a steady increase in research outputs in this area with at least five articles published each year since 2018. *Children and Youth* research also started to surface relatively later than other focus areas. There has also been a steady increase in publications over the years although the trends are not consistent as shown by the trends in Table 2.

**TABLE 2:** Distribution of articles according to year of publication.

Research area	Year of publication								
	1986–1990	1991–1995	1996–2000	2001–2005	2006–2010	2011–2015	2016–2020	2021–2023†	Totals
Assistive technology	0	0	0	0	0	4	25	17	**46**
Children and youth	0	0	0	0	3	13	42	26	**84**
Systems of CBR	2	2	0	2	4	20	30	13	**73**
Development processes	0	3	3	5	15	48	53	31	**158**
Economic empowerment	0	0	0	2	4	11	14	22	**53**
Education	2	0	2	7	8	42	67	61	**189**
Health and HIV and AIDS	0	0	0	2	11	31	45	39	**128**
Holistic wellness	0	0	0	2	4	21	31	9	**67**

**Totals**	**4**	**5**	**5**	**20**	**49**	**190**	**307**	**218**	**798**

† , data collection was concluded in June 2023.

HIV, human immunodeficiency virus; AIDS, acquired immunodeficiency syndrome.

Unlike most of the focus areas, articles on *Systems of CBR* that were retrieved date back to the 1980s and there has been an exponential increase in publications over the years with a more marked increase between 2011 and 2020. Thirty articles were published during this period out of the total of 73. *Development Processes in Africa* has had an exponential increase in number of publications since 1993. It is also one of the focus areas with an early entry onto the research publication platform, but it only started to receive more attention from researchers from 2006.

*Economic Empowerment* came relatively late on the disability research platform in the countries of focus with articles found only dating from 2004. Although the publications are still few, there has been an encouraging steady increase in number of publications focussing on this area from 2004 to 2023. There is also a steep increase in number of publications on *Education*, especially from the year 2011 to date, a period during which 170 were published out of the total of 189. *Education*, across all levels from early to tertiary, is receiving more and more attention from African researchers in AfriNEAD-affiliated countries.

Publications on *Health and HIV and AIDS* are increasing, with three times more publications during period 2015–2019 than those recorded for 2005–2009. Lastly, of all the eight research focus areas, *Holistic Wellness* does not show consistent increase in publications over the years. There is an upward and downward trend in the number of publications over the years since 2006.

### Theme 3: Author affiliations

Most articles were published by authors from a single African country, either at the same institution or in collaboration with others from different institutions in the same country. Collaborations between authors with affiliations in Africa (see Table 3) and those in Europe have the second highest number of articles followed by collaborations across different African countries except for the focus area of *Education*. There are few articles whose authors do not have an African affiliation, although the research focussed on an African context.

**TABLE 3:** Distribution of articles according to the authors’ affiliations.

Research area	Author affiliation									
	African country	Across Africa	Africa/Europe	Africa/US/Canada	Africa/Australia	Europe	Multiple continents	US/Canada	Australia	Totals
Assistive technology	15	2	9	5	2	1	12	0	0	**46**
Children and youth	47	11	17	5	3	1	0	0	0	**84**
Systems of CBR	51	1	11	0	2	2	2	4	0	**73**
Development processes	75	10	36	9	5	10	5	6	2	**158**
Economic empowerment	43	4	4	1	0	0	1	0	0	**53**
Education	146	20	8	9	0	2	1	2	1	**189**
Health and HIV and AIDS	62	7	34	10	3	1	8	3	0	**128**
Holistic wellness	45	2	11	1	2	1	2	3	0	**67**

**Totals**	**484**	**57**	**130**	**40**	**17**	**18**	**31**	**18**	**3**	**798**

HIV, human immunodeficiency virus; AIDS, acquired immunodeficiency syndrome; US, United States.

The same trends shown in the geographical distribution of *Assistive Technology* articles are shown in the affiliations of the authors publishing those articles. Unlike other research focus areas that have a higher number of articles authored by researchers within the same African country, most of the published *Assistive Technology* research is based on collaborations among authors from Africa and those from countries in other continents. The collaborations among African countries on their own are minimal.

*Children and Youth* author affiliations are mostly from within a single African country. Articles published by authors from Africa and Europe collaborating together are more than those co-authored by researchers from different African countries. There are very few articles written jointly by authors from Africa and those from Australia or United States (US) and/or Canada.

Although most of the studies on *Systems of CBR* retrieved were conducted in African countries, author affiliations show a geographical spread that spans across continents. European affiliations dominate as co-authors of articles with authors from Africa within this focus area. More than two-thirds of the articles were written by authors within one African country, and Africa to Africa collaborations are minimal.

There is a higher proportion of authors with affiliations outside Africa in *Development Processes in Africa,* although authors with affiliations from a single African country make almost half the total number of authors. European authors are the most dominant among non-African affiliations within this theme. Collaborations across African countries are fewer when compared to African researchers’ collaborations with authors from the Global North.

More than three quarters of articles under *Economic Empowerment* were published by authors within a single African country. The focus area has low numbers of collaborations across different countries both in Africa and between African researchers and researchers from other regions of the world.

The focus area of *Education* also has more than three quarters of the articles published by authors with an affiliation within a single African country. This is followed by publications where authors from different African countries co-authored. A few articles were published by authors from Africa collaborating with authors based in the US, Canada, Europe or Australia, with combinations of Africa and US and Africa and Europe affiliations being more common.

*Health and HIV and AIDS* has a wide range of author affiliation combinations showing collaborations across Africa. Like most of the other focus areas, collaborations among African countries are scarce. Africa with Europe and Africa with US and/or Canada are the most common collaborations combining African authors with non-African authors. *Holistic Wellness* has mostly authors from a single African country followed by authors from Africa and Europe and very few other categories of collaborations.

## Discussion

Although the results presented in this article are based on an exploratory desktop study, they present a good picture of the current research trends in AfriNEAD-affiliated countries, especially those published by researchers who attend AfriNEAD Conferences. Across the eight research focus areas, the number of publications is increasing exponentially, except for one area of *Holistic Wellness*. The upward trend is more prominent from 2006–2007, coinciding with the period when the UNCRPD was adopted (UN 2006). It was also around the same time when AfriNEAD was established with an agenda to promote UNCRPD principles in Africa using research as a vehicle to inform policy and practice (Kachaje et al. 2014). We envisage that both the adoption of the UNCRPD and the inception of AfriNEAD contributed to the increased focus on disability research in African countries. The launching of the AJOD later in 2011 and publication of special issues following each conference also seem to be contributing factors to the upward trend in the number of publications. African Network for Evidence-to-Action in Disability also contributed to big collaborative international disability research in countries such as Sudan, Malawi, Namibia and South Africa (MacLachlan et al. 2014a) and Uganda, Malawi, Ethiopia and Sierra Leone (MacLachlan et al. 2014b). Several articles were published from these studies.

The different research focus areas are not receiving equal attention from researchers. *Education* and *Development Processes in Africa* are the two focus areas with large numbers of publications, followed by *Health and HIV and AIDS. Economic Empowerment* is the least popular focus area in the research articles retrieved. We acknowledge that it is not possible to explain these trends solely based on the methods used in this desktop exploration. However, we imagine that the increasingly strong focus on inclusive education in many countries (Ohajunwa 2022; United Nations Educational, Scientific and Cultural Organization [UNESCO] 2020) and the impact of the UNCRPD could be major factors pushing research in the two areas with the highest number of publications, respectively. The lack of focus on *Economic Empowerment* could reflect the negative attitudes of society towards the right of persons with disabilities to occupy space in the labour market. It could also be that disability researchers focus on areas related to their professional backgrounds and there are not many professions like occupational therapy that are employment-specific. To understand these trends better, we recommend research that explores factors that determine the reasons researchers tend to focus on certain areas of the lives of persons with disabilities and not others.

*Assistive Technology*, although a latecomer in disability research and only included as a standalone research focus area at AfriNEAD Conferences from 2017, shows significant increase in the number of published articles. It however shows less of single African or Africa to Africa authored articles when compared to other focus areas. The launching of the Global Cooperation on Assistive Technology (GATE) project (WHO 2013) seems to have elicited strong international collaborations in this area. This initiative by the WHO followed global concerns that have risen in recent years pertaining to assistive technology for persons with disabilities and its pivotal role in enabling access to all the other rights and services (Mji & Edusei 2019; Visagie et al. 2022). While drawing from the lessons learned from the international research community, African researchers need to prioritise context relevant issues like adaptation of assistive technology and products and production of technology and products that are suitable and acceptable in the local and indigenous contexts. It is also important that assistive technology should be geared towards improving functionality, participation and social integration of persons with disabilities in Africa to ensure that they are not left behind (Panda 2024). This requires more collaboration and shared experiences among researchers within Africa who live and work in these contexts.

Collaborations are common among authors within the same African country and seldom across different African countries. International collaborations are common among authors from an African country co-authoring with authors from the Global North. One would expect to see more collaborations between African countries compared to the North-South collaborations given the opportunities for networking that AfriNEAD provides (Bezzina 2018; Dwadwa-Henda 2023) but this is not the case. The fact that most academic institutions offering disability studies are in the Global North suggests that younger African researchers who study abroad write their articles with supervisors and other colleagues from that region, which might be one plausible explanation to these findings. Additionally, the fact that African researchers usually rely on research funding bodies from the Global North might mean that the North-South collaborations are preferred better than South-South collaborations. These North-South collaborations can be beneficial as the researchers share knowledge and expertise from the different contexts, but they might perpetuate the power imbalances of Global South researchers being passive recipients of theories and research methodologies from the North as argued by previous scholars (Ned, Dube & Swartz 2022). African governments, businesses and research funders need to prioritise funding disability research to promote more independence among African researchers (Bezzina 2018). There is also a need for South-South collaborations, which seem to be absent from the findings of this review. South–South collaborations have the potential to encourage and promote ‘sharing of problems and sharing of solutions’ (Kerr-Muir, Lehasa & Zondervan 2017), given some of the contextual similarities among countries located within the Global South.

South Africa is by far the highest contributor of articles across the eight research focus areas. The fact that it is a country with two academic institutions with units focussing on disability research coupled with the advancement of disability policy in this country makes disability research a priority. Furthermore, having the AfriNEAD secretariat located at an academic institution in South Africa with more conferences having been hosted in this country also gives the country an advantage over the other countries. Established South African disability researchers should be more active in supporting and strengthening the capacity of researchers in other African countries as AfriNEAD is currently doing via DRCWGs. The support should include mentoring novice researchers from other contexts who present at AfriNEAD Conferences to write their articles for peer-reviewed publications. Currently, as established by this study, many articles that have been presented at the AfriNEAD Conferences have not been published in peer-reviewed journals. Although the reasons why these authors are not publishing have not been established, it is possible that they lack the skills to write peer-reviewed publications, especially the younger, first-time presenters.

Countries like Malawi, Ghana and Zimbabwe also show more disability research being published compared to other AfriNEAD-affiliated countries. These countries have also hosted AfriNEAD Conferences. It is important to note that Ghana also has a disability and rehabilitation programme at KNUST. As AfriNEAD Conferences are tabled in more countries, it is hoped that the presence and influence of the network through its presence in the planning and running of the conferences will encourage more published research conducted in these countries.

### Limitations

The use of AfriNEAD Conference abstract booklets, Google Scholar and AJOD could have limited the number of articles identified for the exploration. This was because of the limited time available to conduct the review. A more systematic search covering other databases might have yielded more results. However, we believe that the results give a good indication of the trends, which was the main aim of the desktop exploration. We also acknowledge that there are blurry boundaries across AfriNEAD’s research focus areas and some articles would have been suitable for more than one research focus area. We recommend rethinking the focus areas and breaking down of focus areas that are too broad into more focussed sub-areas. We also recommend reviews that conduct a critical analysis of the research evidence, including other types of research like prevalence studies so that the trends of what is known and what is not known can become apparent to inform future research and policy and practice.

## Conclusion

This article has shed light on the trends of disability research in countries that have membership in AfriNEAD, especially research published by authors attending AfriNEAD Conferences. Although we did not employ rigorous systematic review methods, we can conclude that at the level at which this desktop study was conducted, there are few things that are apparent; there is a constant increase in the number of publications, especially in the past two decades; South Africa is by far the biggest contributor of disability research articles in AfriNEAD-affiliated countries. Other countries like Ghana, Malawi, Zimbabwe and Zambia are contributing more than the rest of the other countries. Collaborations are more in-country and with countries from the Global North and less across African countries. South-South collaborations are rare. Some research focus areas are more popular than others, particularly, *Education* and *Development Processes in Africa*. We see this desktop exploration as a first step for AfriNEAD to get a baseline understanding of disability research in the countries affiliated to the network. Further research is required to understand these trends, to do critical analysis of the current research evidence and to provide evidence necessary to address the identified gaps.

## Footnotes

1 These are countries that have people who are members of AfriNEAD in their own capacity as individuals or as members of organisations focussing on disability issues.

2 See Addendum for details of the six conferences.

## Addendum: Themes of the first six AfriNEAD Conferences and hosting countries

1. 2007: ‘Realising the rights of disabled people in Africa’: Cape Town, South Africa.
2. 2009: ‘The ABC of research Evidence-to-Action: Putting United Nations Convention on the Rights of Persons with Disabilities (UNCRPD) principles into action for a rights-based change’: Cape Town, South Africa.
3. 2011: ‘Building communities of trust: Evidence–to-Action in disability research’: Victoria Falls, Zimbabwe.
4. 2014: ‘Intensifying disability research and practice to achieve MDGs (Millennium Development Goals) in Africa: Our experience and aspirations for the future’: Mangochi, Malawi.
5. 2017: ‘Disability and inclusion in Africa: The role of Assistive Technology’: Kumasi, Ghana.
6. 2020: Virtual: ‘Disability unplugged-Beyond Conventions and Charters: what really matters to persons with disabilities in Africa’: Cape Town, South Africa.
